# Midterm Outcomes for Funnel-EVAR

**DOI:** 10.31083/j.rcm2506224

**Published:** 2024-06-20

**Authors:** Bahadır Aytekin, Serkan Mola, Gökay Deniz, Sinan Özçelik, Hakkı Zafer İşcan

**Affiliations:** ^1^Department of Cardiovascular Surgery, Ankara Bilkent City Hospital, 06800 Ankara, Turkey

**Keywords:** funnel, trombone, wide aortic neck, EVAR, 60 mm length of thoracic endograft

## Abstract

**Background:**

The funnel technique, the hybrid assembly of a thoracic and abdominal aortic endograft, is advantageous for frail patients where efficient oversizing is not possible for infrarenal wide aortic necks over 34 mm. We sought to determine the advantages and disadvantages of the Funnel-endovascular aneurysm repair (EVAR) technique using 60 mm length thoracic endograft.

**Methods:**

This retrospective study included 22 patients, all frail with high comorbidities, who were operated on with the Funnel technique using the 60 mm Lifetech Ankura thoracic endograft, in 7 urgent and 15 elective cases from January 2018. There were no exclusion criteria except having an age <60 years. Primary endpoints were the technical success and early mortality and morbidity; secondary endpoints were late outcomes such as endoleak, migration, late open surgical conversion, successful sac shrinkage, and enlargement at the infrarenal aortic neck diameter.

**Results:**

The patients’ mean age was 72.6 ± 7.3 years (62–86 years), with a mean aneurysm diameter of 83.2 ± 16.8 mm and mean infrarenal aortic diameter of 38.7 ± 2.4 mm. There was no early mortality. Technical success was 100%. 21 standard bifurcated and one aorto-uni-iliac abdominal endograft were deployed. The mean fluoroscopy time was 14.3 ± 5.2 minutes. Mean follow-up was 32.8 ± 19.6 months, with no endovascular complications. There was no Type-1a or Type-3 endoleak, migration, infrarenal aortic neck diameter enlargement, or aneurysm sac enlargement. During the follow-up, three patients died, but there was no aneurysm-related mortality.

**Conclusions:**

Funnel-EVAR is effective and safe for patients with a wide infrarenal aortic neck diameter when assessing midterm outcomes. Therefore, it should be part of the armamentarium of a vascular surgeon in patients with wide aortic necks >34 mm.

## 1. Introduction 

Endovascular procedures have become the treatment of choice for all anatomically suitable aneurysm patients, since it is a less invasive technique, and has resulted in decreased morbidity and mortality compared to open aortic repair. Endovascular aneurysm repair (EVAR) is the dominant treatment for infrarenal abdominal aortic aneurysms (AAAs) for frail patients, and now accounts for 70–80% of all repairs [1].

Hostile neck anatomy, especially wide or ectatic aortic necks, are the most common inhibitory factor for endovascular procedures. Large infrarenal aortic necks treated with standard EVAR are more complicated than the smaller necks; however, there are still ongoing debates about this topic [2, 3, 4, 5]. Since efficient oversizing is not possible for infrarenal wide aortic necks over 34 mm, most undergo open surgical repair with high morbidity and mortality. Fenestrated, branched endograft, chimney, or Surgeon Modified Fenestrated Stent Graft (SMFSG) place the proximal sealing side upwards to the abdominal visceral aorta or revascularizing the visceral branches with a parallel graft technique [6]. In such cases, as an endovascular solution, the so-called “Funnel” or “Trombone” technique may be used [7, 8, 9, 10, 11, 12, 13, 14]. The Funnel technique is the hybrid assembly of a thoracic and abdominal aortic endograft, and is especially advantageous to the frail patient.

There are less than fifty cases reported [11], mostly case reports or case series [7, 8, 9, 10, 11, 12, 13, 14], performed with aorto-uni-iliac or bifurcated abdominal endografts with a hybrid assembly of a 10 cm thoracic endograft. We started to perform this technique as a bail-out procedure in 2018, and after being confident about the outcomes [9], we began to perform them on patients with increased co-morbidities. We present one of the largest studies of Funnel-EVAR 60 mm Lifetech Ankura thoracic and abdominal bifurcated endograft. We discuss the pros and cons of this technique with midterm outcomes, and review the current literature to determine the role for the Funnel-EVAR in the treatment of this patient cohort.

## 2. Materials and Methods

### 2.1 Study Design

Between January 2018–January 2024 we operated on 22 patients who had infrarenal wide aortic necks over 34 mm. The same cardiovascular surgical team operated on all patients in an angiography suite. There were no exclusion criteria except having an age <60 years. The institutional ethics board approved the study protocol (Date/No: 09.12.2020/E1-20-1385). The study was conducted following the principles of the Declaration of Helsinki. All patients were in American Society of Anesthesiologists (ASA) class III–IV status, and fifteen were symptomatic. Four patients were under cancer therapy. Three patients had previous EVAR and experienced migration, and one had a leg thrombosis with ischemia. Preoperative data are shown in Table 1.

**Table 1.** **Demographics of patients**.

Features	n (%) or mean ± SD
Age, years	72.6 ± 7.3
Male gender	22 (100)
Comorbidities	
Hypertension	15 (68.1)
Hyperlipidemia	12 (54.5)
Diabetes mellitus	5 (22.7)
Coronary artery disease	14 (63.6)
Chronic obstructive pulmonary disease	12 (54.5)
Malignancy	4 (18.2)
Coronary artery bypass grafting	5 (22.7)
Smoking habit	18 (81.8)
Peripheral artery disease	3 (13.6)
Aneurysm diameter, mm	83.2 ± 16.8 mm

SD, standard deviation.

**Fig. 1.**
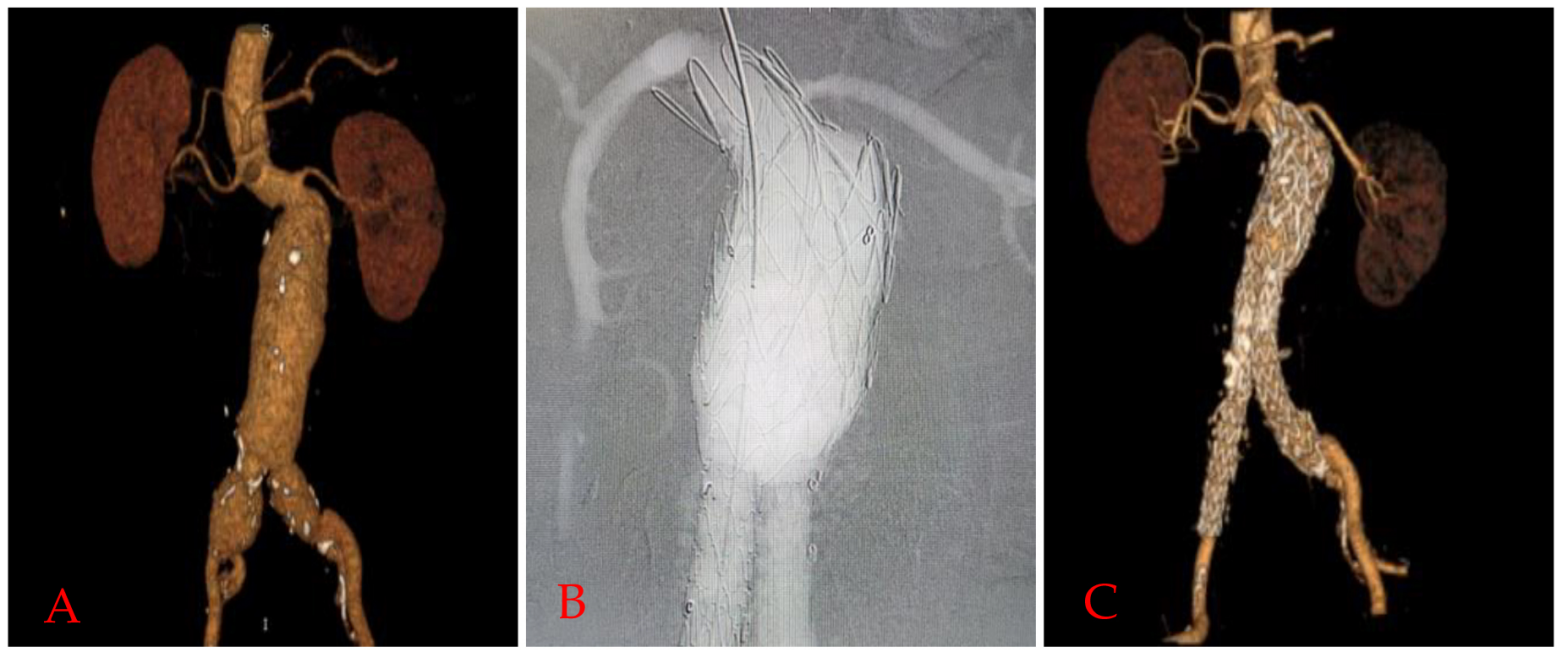
**Funnel EVAR application in a patient with hostile neck. **Preoperative 3D reconstruction image of computed tomographic angiography scan (A), completion angiography image (B), and 24. Month computed tomographic angiography (CTA) controls (C) of a >${\mathrm{60}}^{\circ }$ angulated Funnel-EVAR with no endovascular complication. EVAR, endovascular aneurysm repair; 3D, three dimensional.

All patients had an infrarenal aortic neck over 35 mm on computed tomographic angiography (CTA). 4 patients had conical necks, and two patients had severe angulation >60 degrees (Fig. 1A–C).

**Fig. 2.**
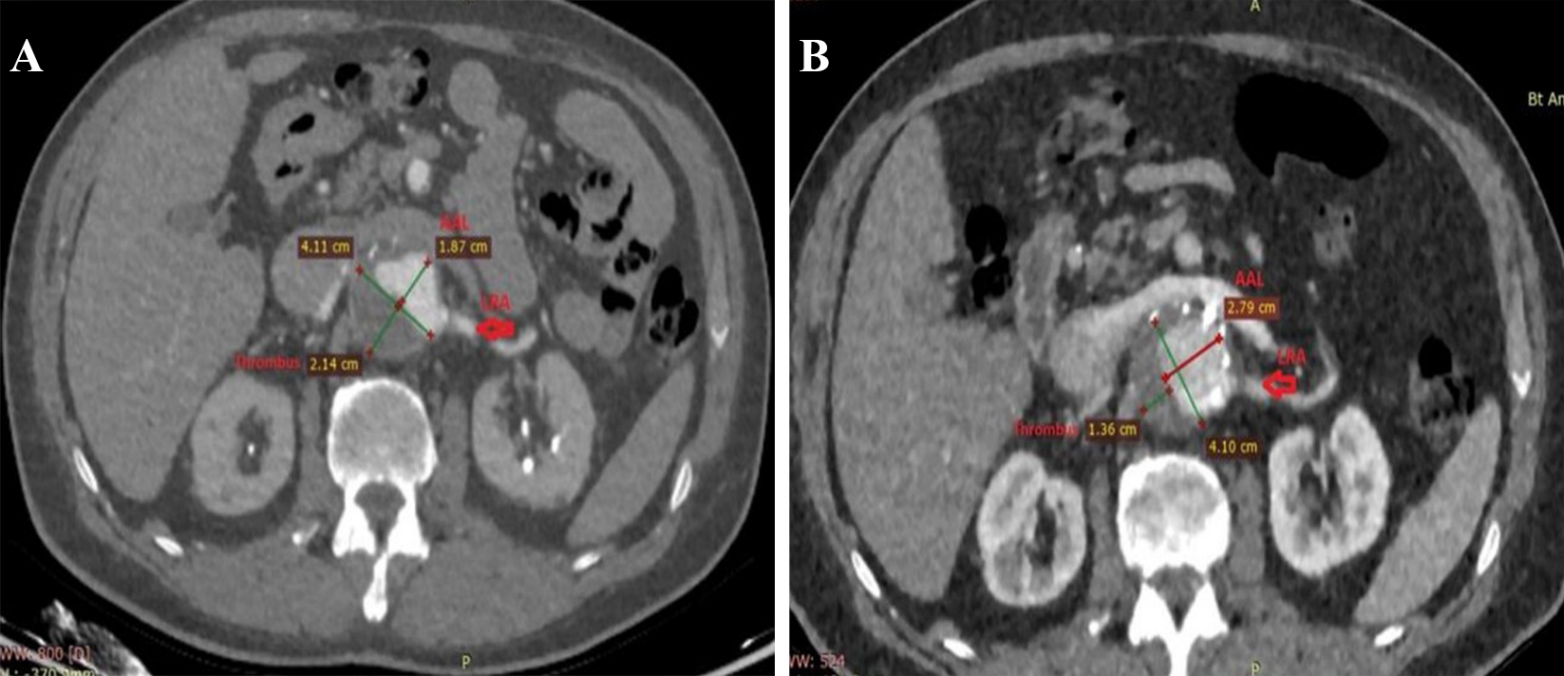
**Remodeling of thrombus and true lumen in the aneurysm after Funnel EVAR.** Preoperative thrombus burden at the infrarenal neck (A) and postoperative diminished thrombus burden and enlarged active lumen with no infrarenal neck enlargement at 48 months CTA control (B). EVAR, endovascular aneurysm repair; CTA, computed tomographic angiography; AAL, aortic active lumen; LRA, left renal artery.

One patient also had a contained rupture in the thoracic segment. There was a heavy thrombus burden in 5 patients circumferentially involving nearly 25-50% of the neck (Fig. 2A,B).

### 2.2 Surgical Technique

**Fig. 3.**
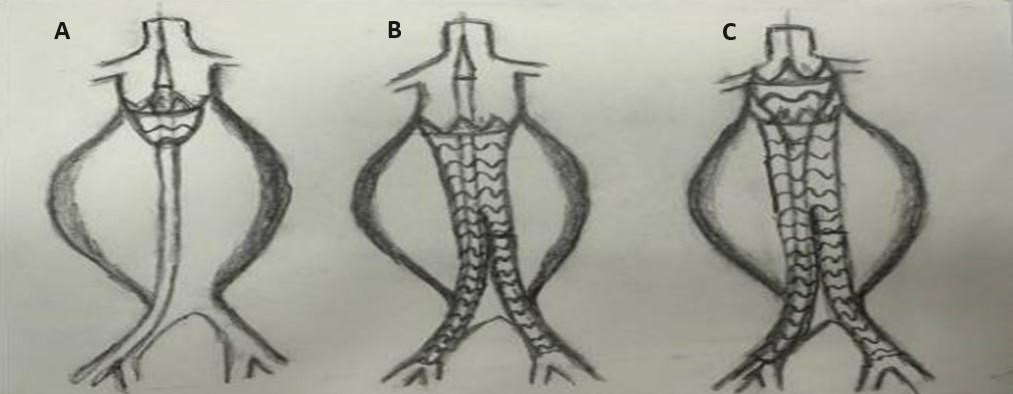
**Technical details and how-to-do algorithm for Funnel-EVAR.** (A) Abdominal endograft was deployed 2 or 3 cm below the lowest renal artery. (B) Stabilization at the distal landing zone whether common or external iliac arteries. (C) Finally deploying the funnel part with a 60 mm length of thoracic endograft. EVAR, endovascular aneurysm repair.

Standard abdominal or thoracic endografts of any brand may be used for this technique. Since the aortic neck was over the size of efficient oversizing for an abdominal endograft, the largest sized abdominal endograft (34 mm Lifetech Ankura (Shenzhen, China) or 36 mm Medtronic Endurant II (Santa Monica, CA, USA)) was used. For the funnel side, since the maximum diameter is 46 mm in size, 10–20% of oversizing with 60 mm length of a Lifetech Ankura Thoracic endograft was deployed for the proximal neck. As described in our previous study [9] (Fig. 3), every step is the same as for a standard EVAR procedure. For all patients, open surgical femoral access was utilized bilaterally. After placing the guidewires and parking the pigtail for renal visualization, the abdominal endograft was deployed first just 2–3 cm below the lowest renal artery, and the endograft was stabilized at both distal landing zones (mostly commonly the iliac arteries) to avoid migration before deploying the thoracic part. Completion angiography was always performed.

### 2.3 Definitions and Endpoints

Technical success was defined as no Type 1 or 3 endoleak and total exclusion of the aneurysm sac on completion angiography. Successful sac shrinkage and positive remodelling were defined as over a 5 mm decrease in maximum aneurysm diameter. Primary endpoints were the technical success, and early mortality and morbidity; secondary endpoints were late outcomes such as endoleak, migration or late open surgical conversion, successful sac shrinkage, and enlargement at the infrarenal aortic neck diameter. All patients were followed up with Colored Doppler Ultrasound (CDUS) every 6 months and Multislice CTA annually.

### 2.4 Statistical Analysis 

Normally distributed continuous variables were expressed as mean values ± standard deviation (SD). Categorical variables were expressed as numbers and percentages. Kaplan-Meier analysis was used to assess the survival outcome. All statistical analyses were performed using the SPSS statistical software (SPSS for Windows 15.0, Inc., Chicago, IL, USA).

## 3. Results

The mean age of the patients was 72.6 ± 7.3 years (62–86 years), the average maximum aneurysm diameter was 83.2 ± 16.8 mm (69–117 mm), and the average infrarenal aortic diameter was 38.7 ± 2.4 mm (35.5–41.1 mm). There were 6 urgent cases, and the rest were elective ASA III-IV patients with high comorbidities.

There was no early mortality in this patient cohort. Technical success was 100%. 21 standard bifurcated and one aorto-uni-iliac abdominal endograft were deployed. A 60 mm length thoracic endograft was deployed for all patients. Carbon dioxide-guided angiography was used in one patient because of moderate renal insufficiency. In three patients, Funnel-EVAR was performed through migrated EVARs.

All previous endografts were bifurcated endografts. All procedures were performed under general anesthesia. The average fluoroscopy time was 14.3 ± 5.2 minutes, and 60 ± 12.5 mL of contrast media was administered and 50% diluted in all standard EVAR procedures.

**Fig. 4.**
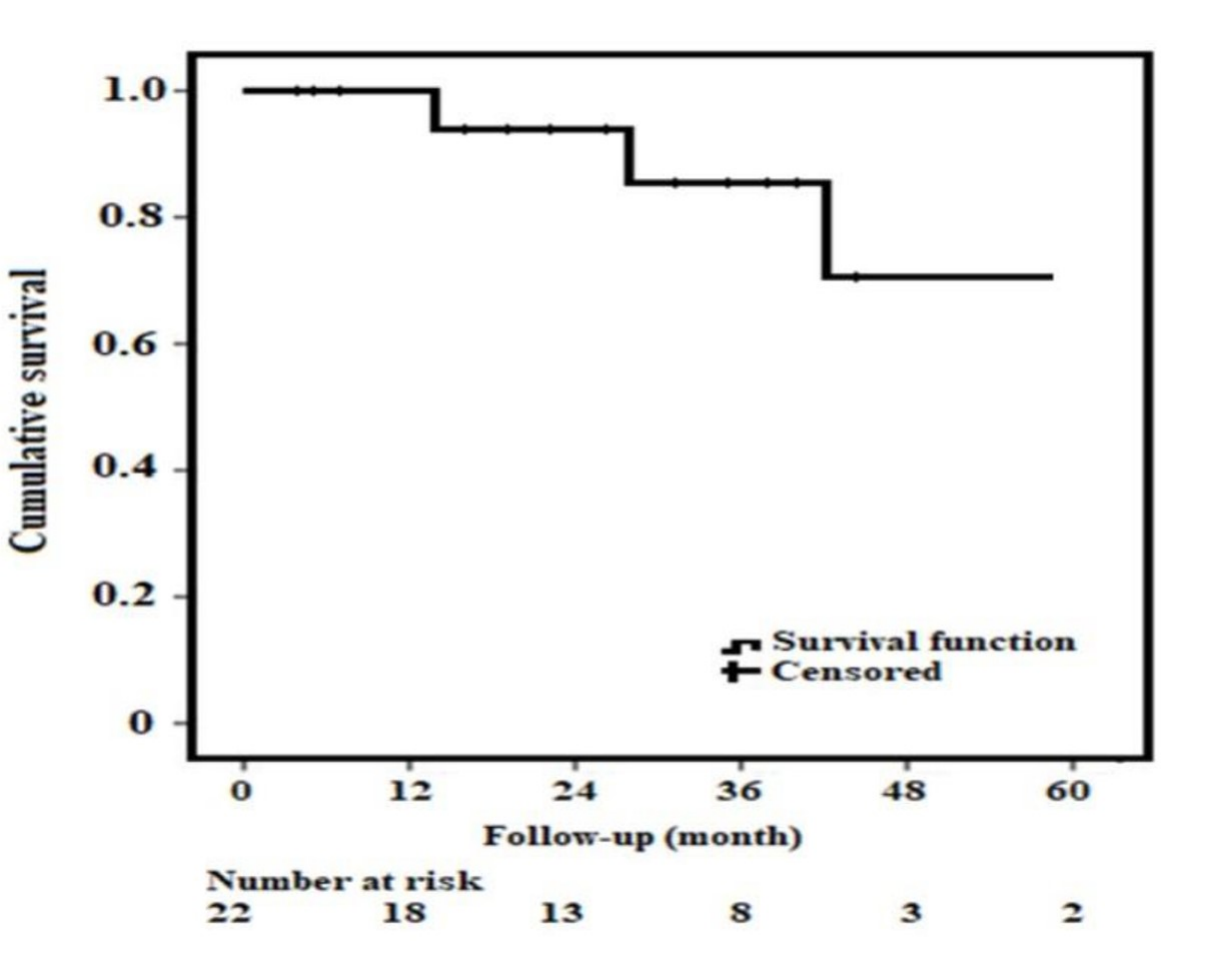
**Kaplan-Maier Survival Analysis. **Cumulative survival for patients with Funnel EVAR and patients at risk. EVAR, endovascular aneurysm repair.

Median follow-up was 32.8 ± 19.6 (4–62) months, with no endovascular complications. There were no Type 1a or Type 3 endoleaks, no migration, no infrarenal aortic neck diameter enlargement, no aneurysm sac enlargement. Successful aneurysm sac shrinkage was achieved (>5 mm) after six months in half of the patients, the rest of the sacs were stabilized. In the follow-up period, three patients died: one with lung cancer in the 14th month, the second from cardiac disease in the 28th month, and the last because of endograft infection and sepsis postoperatively in the 42nd month. The patient was hospitalized twice; after unsuccessful antibiotic therapy, complete graft extirpation and aortobiiliac bypass with a silver-coated surgical dacron graft was performed. The patient survived the operation; however, he died due to sepsis and multiorgan failure on the second postoperative day. Fig. 4 shows the Kaplan-Meier cumulative survival curve for the Funnel-EVAR patients.

## 4. Discussion

The survival benefit of endovascular procedures may be explained by a decreased rate of cardiac events compared to open surgery in frail and elderly patients. EVAR used with the funnel technique (FT-EVAR), first described by Zanchetta* et al*. [7], consists of placing a bifurcated abdominal endograft as close as possible to the native aortic bifurcation followed by a 100 mm thoracic endograft as a proximal cuff. We have been performing the Funnel-EVAR technique since January 2018. Initially it was used as a bail-out procedure. The first seven patients were all symptomatic and frail ASA class IV patients operated on urgently. After 2020, we began to perform this technique on elective patients when we realized the dilated aortic neck was not enlarging. Surprisingly, there were no endovascular complications with this modified Funnel technique [9]. In our experience, Funnel-EVAR is practical, always available, and devoid of complications, at least during the midterm follow-up period. With our modified technique, using a 60 mm length of thoracic endograft, we deploy the abdominal endograft 2–3 cm below the lowest renal artery and then stabilize the system at the distal part bilaterally, with the 60 mm funnel graft.

The lowest renal artery to aortic bifurcation distance is the main limitation of this technique. For this reason, as almost all thoracic endografts are 100 mm in length, Endologix AFX (Endologix, Irvine, CA, USA) short main bodies [10, 12], aorto-uni-iliac abdominal endografts [8] are deployed as close as possible to the aortic bifurcation. Bastiaenen* et al*. [8] has shown that to perform this technique, the distance from the lowest renal artery to aortic bifurcation should be at least 100 mm for an aorto-uni-iliac endograft and 130 mm for a bifurcated standard endograft. For this reason, our solution was to use a 60 mm length of LifeTech Ankura Thoracic endograft [9, 10, 11, 12, 13, 14, 15, 16]. We think shorter thoracic endografts, such as those measuring 60 mm length, offer a potential advantage in preventing late-type IA or III endoleaks. This advantage stems from 30 mm land of thoracic endograft deployed at the proximal segment and the rest 30 mm segment for overlap which remains unaffected by dominant sideway forces due to its design, thereby enhancing its resistance to endoleaks.

De Bruijn* et al*. [12] reported five patients with an AFX unibody platform (Endologix, Irvine, CA, USA) which has a short main body. They also used endo-anchors if needed. Cooper *et al*. [10] also used the same AFX unibody platform for seven patients and reported one late Type III endoleak. The sideway movements may be the reason for Type III endoleaks, as we reported in our AFX experience [16] for longer thoracic segments. However, in our technique (Fig. 3), using the Lifetech Ankura 60 mm length of thoracic endograft, we were able to place the endograft 2–3 cm below the lowest renal artery. Hence, the funnel part was shorter, jammed between the blood flow direction, and strongly stabilized the abdominal endograft through the aortic neck. 2–3 cm of overlap was also crucial for sufficiently opening the thoracic endovascular aortic repair (TEVAR) endograft.

A review of the literature regarding this technique was performed by Amico* et al*. [11]. In 2021, they found 32 reported cases of Funnel-EVAR with a follow-up ranging from 2 to 84 months (mean 22 months). There was one conversion to open repair due to graft migration (3%) and one aneurysm-related death. There were four late Type Ia endoleaks (13%), resulting in no aneurysm-related deaths [11]. In an effort to decrease endoleaks, some authors have advocated Endoanchor (Aptus system – Medtronic) fixation [14, 17, 18]. The Aneurysm Treatment Using the Heli-FX Aortic Securement System (ANCHOR) demonstrated the protective effects of endoanchors on aortic neck dilatation (AND) or enlargement. Endoanchors were thought to fix and stabilize the endograft and prevent neck enlargement [17, 18]. Monahan* et al*. [17] and Ribner and Tassiopoulos [18] reported that when the aortic neck diameter reaches the nominal endograft diameter, the endoanchors stabilize the aortic wall, preventing further neck dilatation.

Wide necks over 28 mm tend to have more Type Ia endoleaks and require late interventions [2, 3, 4]. Patients with wider proximal aortic necks also have shorter necks and larger aneurysm diameters. This may be the reason for the higher morbidity rates. We think that the most complex issue for this technique is placing the endograft at the diseased aortic segment. In the ageing aorta, there is ongoing aneurysmal disease as the aortic wall structure is negatively altered in a progressive manner. In our experience, we did not face any neck enlargement and no aneurysm-related complications. Tassiopoulos* et al*. [19] reported that small necks appear to be at higher risk for subsequent dilatation whilst matching the size of the endograft. Amongst the different Funnel-EVAR techniques, although it depends on the availability of the endograft sizes, using a longer thoracic endograft may create the possibility of sideway movements and result in Type III endoleaks [10, 12, 16]. Using aorto-uni-iliac endografts may alter the patency of extra-anatomic bypass grafts [8]. After standard EVAR, AND may develop with an incidence of 20–28% at two years and up to 43% after open surgical repair [17, 18, 19, 20]. Ongoing aneurysmal disease or the radial force of the oversized endograft may be the reason for this complication. No neck enlargement was demonstrated in our midterm follow-up period (Fig. 2A,B).

An important technical detail for successful outcomes in Funnel-EVAR is the stabilization of the endograft at the iliac arteries, either common or external. Tortuosities of the iliac vessels or losing stabilization may result in endoleaks or migration. Another treatment modality or bilateral stabilization of the endograft at the external iliac arteries should be considered if needed because of a short common iliac or tortuosity.

Compared to complex endovascular repairs such as fenestrated, branched, or chimney techniques, Funnel-EVAR is readily available. Custom-made endografts have availability problems, are more expensive, and require longer waiting periods. Funnel-EVAR is technically easier and results in less Type Ia endoleaks (gutter endoleaks) than the chimney technique. Moving the proximal side to the abdominal visceral aorta with surgeon-modified fenestration can occlude the branches of vital organs, or require branch stenting with its own complications. The advantages of Funnel-EVAR is that it is always available, is easy to deploy, and requires no advanced endovascular skills. When compared to open surgical repair, endovascular techniques have lower morbidity and mortality in these frail patients.

Ectatic aortic necks also frequently have a thrombus burden. In our experience, a thrombus did not influence the technical success of EVAR. We did not perform any ballooning procedures to the thrombus at the infrarenal neck. The thrombus was probably thinner and diminished in the CTA controls because of radial force, and we called it the “pillow effect” (Fig. 2A,B). Close monitoring is mandatory in the presence of neck thrombus due to concerns regarding embolization. Shintani* et al*. [21] reported that neck thrombus did not affect the incidence of Type Ia endoleak or migration. However, it was significantly associated with thromboembolic complications such as distal embolization and renal dysfunction [21].

**Fig. 5.**
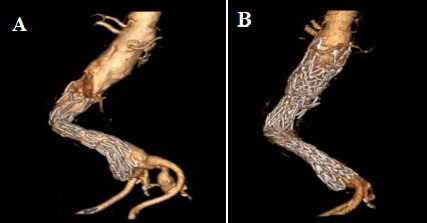
**Treatment of migrated endograft with Funnel EVAR.** Preoperative CTA of a migrated index EVAR procedure (A) and postoperative 6th month CTA control with no complication (B). EVAR, endovascular aneurysm repair; CTA, computed tomographic angiography.

Hybrid utilization of endografts prevents endoleaks and migration when shorter thoracic endografts are utilized. Also, reintervention with Funnel-EVAR is a potential alternative solution for migrated EVAR (Fig. 5A,B).

**Fig. 6.**
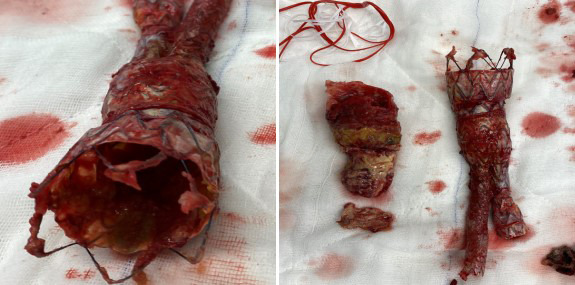
**The durability of overlapping endografts.** Extirpated thoracic and abdominal endograft, not separated even though it was forced to pull out from the native aorta. Removing the thoracic part from the native aortic neck was easier because there was no active fixation system.

While examining our patient’s extirpated endograft, the funnel part was not easily taken out from the abdominal part (Fig. 6).

Funnel-EVAR is practical, always available, and there is nearly no technical limitation with the 60 mm length of the thoracic segment.

This study is limited by its retrospective design, single center experience and small patient cohort. However this study is thought to be the largest cohort in this patient group with a bifurcated abdominal endograft. Furthermore, we performed no comparisons with other techniques including open surgical repair. Long-term follow up data will be necessary to further define the role of Funnel-EVAR in this high risk patient cohort.

## 5. Conclusions

In conclusion, Funnel-EVAR appears to be safe and effective for patients with wide infrarenal aortic neck diameter based on midterm outcomes. It provides a practical solution for this complex patient cohort and should be kept in the vascular surgeon’s armamentarium.

## Data Availability

The datasets used and/or analyzed during the current study are available from the corresponding author on reasonable request.
